# Combined treatment with antitoxin and 3,4-diaminopyridine improves survival outcomes after lethal botulinum neurotoxin challenge

**DOI:** 10.1186/s10020-025-01316-0

**Published:** 2025-07-28

**Authors:** Sean W. O’Brien, Brieana M. Gregg, Adhirath Bollapragada, Patrick M. McNutt

**Affiliations:** 1 Wake Forest Institute for Regenerative Medicine, Wake Forest University School of Medicine, Winston-Salem, NC 27101 USA; 2 Department of Microbiology and Immunology, Wake Forest University School of Medicine, Winston-Salem, NC 27101 USA

**Keywords:** Botulinum neurotoxin, Botulism, Aminopyridine, 3,4-diaminopyridine, Muscle paralysis, Neuromuscular junction, Preclinical models, Drug delivery

## Abstract

Botulinum neurotoxins (BoNTs) are the most potent toxins known, causing life-threatening flaccid paralysis by blocking acetylcholine release at neuromuscular junctions. Clinical botulism results in respiratory failure, requiring prolonged artificial ventilation for survival. The only specific therapy is antitoxin, which neutralizes circulating toxin but cannot affect toxin within neurons, resulting in a narrow therapeutic window. Due to its high potency and lack of treatment options, large-scale BoNT exposures present profound risks to human life. Thus, there is an urgent need for rapidly acting symptomatic therapies that can reverse respiratory paralysis and sustain survival until resolution of toxin effects. We previously identified the FDA-approved, voltage-gated potassium channel blocker 3,4-diaminopyridine (3,4-DAP) as a fast-acting symptomatic treatment in preclinical botulism models. Here, we expand upon those findings by evaluating continuous infusion of 3,4-DAP in rats exposed to a typical range of BoNT/A doses. Infusion of clinically relevant doses of 3,4-DAP improved survival, reversed respiratory paralysis, and rapidly alleviated clinical signs at toxin doses substantially higher than previously studied. Therapeutic efficacy of 3,4-DAP correlated inversely with toxin exposure, consistent with its proposed mechanism of enhancing acetylcholine release from residual functional synaptic vesicle pools. 3,4-DAP treatment remained effective even when initiated at advanced stages of botulism, when antitoxin monotherapy provided no benefit, significantly extending the clinical treatment window. Combining 3,4-DAP infusion with antitoxin had robust effects on clinical outcomes, reversing clinical symptoms and improving survival compared to either treatment alone. Monitoring of body temperature further revealed that significant hypothermia precedes overt clinical symptoms, providing a novel biomarker of intoxication and treatment efficacy, as toxin-induced hypothermia resolved within hours after 3,4-DAP administration. Collectively, these findings provide robust preclinical evidence supporting clinical translation of 3,4-DAP as a symptomatic reversal agent for botulism, potentially transforming clinical management strategies for this lethal neurotoxin-induced disease.

**Supplementary Information:**

The online version contains supplementary material available at 10.1186/s10020-025-01316-0.

## Introduction

Botulinum neurotoxins (BoNTs) are highly toxic, neuroparalytic proteins that present a significant threat to public health. To date, over 40 BoNT variants have been identified and classified into seven serotypes (BoNT/A-G) based on genetic and antigenic differences (Pirazzini et al. 2017). All BoNTs are heterodimeric toxins composed of a 50 kDa light chain, which cleaves neuronal SNARE proteins, and a 100 kDa heavy chain, which facilitates neuron-specific binding, internalization, and translocation into the neuronal cytosol (Simpson 2004; Montal 2009; Burgen et al. 1949). BoNTs are the most toxic substances known, with median lethal doses as low as 0.2 ng/kg, depending on the serotype and route of exposure (Pirazzini et al. 2017). The extreme toxicity of BoNT is attributable to several properties, including a high avidity for presynaptic receptors and the efficient proteolysis of SNARE proteins required for acetylcholine release. The ensuing blockade of neuromuscular transmission manifests as a descending flaccid paralysis within 24 h that typically leads to respiratory arrest within 72 h (Lindstrom and Korkeala 2006; Witoonpanich et al. 2010). Survival after a lethal exposure requires artificial ventilation and parenteral nutrition until respiratory paralysis resolves, which can require weeks to months. Death typically occurs from early respiratory failure or complications related to prolonged intensive care, such as ventilator-associated pneumonia or deep vein thrombosis (Sobel 2005).

If untreated, botulism has a mortality rate exceeding 60% (Dembek et al. 2007). The only approved treatment for botulism is post-exposure prophylaxis with equine-derived immunoglobulin antitoxins, which terminate exposure by binding and neutralizing BoNT in the bloodstream. However, antitoxins cannot neutralize toxin already bound to neuronal receptors or internalized into neurons. As a result, antitoxin treatment has a narrow therapeutic window, and 70% of botulism patients given antitoxin after symptomatic emergence still require artificial ventilation and enteral nutrition for survival (Richardson et al. 2019; Yu et al. 2017). Some BoNT serotypes can cause paralysis that lasts for weeks to months, necessitating prolonged supportive care and increasing the risk of ventilator-associated complications (Sobel 2005). Despite decades of effort, developing therapeutics against botulism has proven challenging and there is little appetite for immunization given the increasing number of clinical indications for BoNT-based therapeutics. Although recent research has identified intracellular treatments aimed at blocking or clearing the light chain from nerve terminals, these antidotes are still in early development (McNutt et al. 2021; Miyashita et al. 2021). Moreover, they do not address the initial muscle weakness and paralysis that are the earliest clinical signatures of botulism and which persist until damaged SNARE proteins are naturally replaced. The limitations of current and emerging therapies underscore the urgent need for fast-acting treatments that can rapidly reverse respiratory weakness and sustain survival until definitive care is available. This need is particularly exigent in mass casualty scenarios or for victims in austere circumstances with limited access to rapid transportation to definitive care, such as military personnel. Ideally, such treatments could also improve respiratory function throughout the course of disease, accelerating functional recovery from botulism and reducing the risk of ventilator-associated complications.

The FDA-approved drug 3,4-diaminopyridine (3,4-DAP; IUPAC amifampridine) is a fast-acting, symptomatic treatment for botulism. 3,4-DAP reversibly blocks voltage-gated potassium channels involved in action potential repolarization, prolonging action potential duration and enhancing acetylcholine release (Thomsen and Wilson 1983; Kirsch and Narahashi 1978; Ojala et al. 2021). Mechanistic studies in primary neuron cultures and phrenic nerve-hemidiaphragm preparations demonstrate that 3,4-DAP increases acetylcholine release from intoxicated nerve terminals, functionally treating the neurophysiological pathology in botulism (Beske et al. 2017; Bradford et al. 2018). In mice, a single administration of clinically relevant doses of 3,4-DAP rapidly reverses acute respiratory paralysis at advanced stages of terminal botulism, significantly improving ventilation, reversing respiratory acidosis, increasing activity, and alleviating toxic signs (McClintic et al. 2024; Vazquez-Cintron et al. 2020). Repeated injections of 3,4-DAP significantly extend survival in mice (Vazquez-Cintron et al. 2020) and rats (Machamer et al. 2022) challenged with up to five median lethal doses (LD_50_) of BoNT/A, suggesting that long-term administration of 3,4-DAP may sustain survival until botulism symptoms resolve. Consistent with this hypothesis, continuous infusion of 3,4-DAP via subcutaneous catheter had antidotal efficacy in rats challenged with 2.5 LD_50_ BoNT/A (Machamer et al. 2022). Symptomatic rebound was not observed when 3,4-DAP infusion was stopped after clinical signs resolved, providing the first demonstration of a small-molecule treatment for botulism. In contrast, withdrawing 3,4-DAP infusion while rats still exhibit toxic signs of botulism causes the re-emergence of clinical signs and a rapid progression to respiratory paralysis and death. These data show that the antidotal efficacy of 3,4-DAP requires sustained treatment until botulism symptoms resolve. Notably, in these studies, 3,4-DAP was administered at infusion dose rates that produce steady state plasma levels comparable to clinical dosing, suggesting direct translational potential.

These foundational studies support the clinical development of 3,4-DAP as a reversal agent for botulism symptoms. Here we build upon these findings by evaluating the symptomatic and antidotal effects of 3,4-DAP infusion in rats challenged with 1.8–10 LD_50_ BoNT/A, representing the typical range of foodborne BoNT/A doses (Pirazzini et al. 2017). BoNT serotype A is the predominant etiological agent of foodborne and iatrogenic botulism and among the most hazardous serotypes (Pirazzini et al. 2017). The benefits of combined treatment of 3,4-DAP with antitoxin are also explored, recapitulating the most likely clinical treatment scenario. Finally, the clinical benefits of delayed treatment with 3,4-DAP are tested at times when antitoxin is ineffective, evaluating the ability of 3,4-DAP to compensate for the narrow treatment window of antitoxin. Collectively, our findings further support the clinical potential for 3,4-DAP as a symptomatic reversal agent for botulism.

## Methods

### Animals

Male Sprague–Dawley rats (350–425 g; Charles River Laboratories) were group-housed, maintained on a 12 h diurnal cycle and provided a standard diet with regular enrichment and water ad libitum. Rats were implanted with subcutaneous catheters (Charles River Laboratories, Raleigh, NC) attached to one-channel rodent ports (Instech Laboratories, Plymouth Meeting, PA) that were surgically secured between the scapulae. Catheters were flushed every 3–5 d with 0.5 mL saline to ensure patency. For intoxication, rats were randomly assigned to groups and administered BoNT/A diluted in phosphate-buffered saline with 0.2% gelatin (Millipore Sigma, St. Louis, MO) in a volume of 250 µL by tail vein injection. Rats were euthanized with 3% isoflurane followed by decapitation. In some experiments, rats were implanted with commercially available RFID microchips including temperature probes (Unified Information Devices, Lake Villa, IL) and read using a handheld RFID scanner (Unified Information Devices).

### Determination of BoNT/A intravenous LD_50_ in rats

Botulinum neurotoxin serotype A (10 µg/mL in 30 mM sodium acetate, 2 mg/ml gelatin, 3 mg/ml bovine serum albumin, pH 4.2) was purchased from Metabiologics (Madison, WI) and stored at 4 °C. BoNT/A was diluted to working concentrations in PBS with 0.2% gelatin (ThermoFisher, Waltham, MA). The specific activity of each lot of toxin was determined using a stagewise, adaptive design, as previously described (Machamer et al. 2022). Survival was recorded at daily intervals for up to 5 d and the intravenous LD_50_ was calculated by simple logistical regression. Stagewise dosing continued until the 95% CI was within ± 12.5% of the calculated LD_50_ values. The rat intravenous LD50 values of the three lots of toxins used in this study ranged from 175–245 pg/g. For simplicity, all toxin doses are referred to in terms of rat intravenous LD_50_.

### 3,4-DAP efficacy studies

For subcutaneous infusions, infusion ports were connected to syringes via tethers (Instech) that run through snap-in spring swivel arms (Instech) mounted on each cage. Rats were acclimated to infusion tethers starting 1 d prior to BoNT/A intoxication. On the day of intoxication, rats were weighed and intoxicated with target doses of BoNT/A via tail vein injection. Once the plurality of animals within each study exhibited clinical signs of botulism, infusion of saline or 3,4-DAP was started at 333 µL/h using programmable six-port syringe pumps (World Precision Instruments, Sarasota, FL). Saline and 3,4-DAP infusates were prepared daily using the weights measured at time of intoxication and loaded into 10 mL syringes. At daily intervals, the infusion pump was stopped for less than 2 min so depleted syringes could be replaced with syringes containing fresh infusate, and the infusion pump was restarted. Toxic signs and mortality were recorded at ≤ 6 h intervals from 0–5 d and at daily intervals thereafter. To mitigate malnutrition, oral gavage of liquid nutrition (Boost Very Vanilla complete nutritional drink; Nestlé Health Science, Vevey, Switzerland) was administered twice daily through a ball-tip stainless steel feeding needle (Cadence Science, Staunton, VA) for a total daily administration of 5 mL/kg. Oral gavage volumes were calculated using the weights measured at time of intoxication. If rats drank from the feeding needle prior to gavage, gavage was not performed. Oral gavage started no earlier than 6 d and continued until voluntary consumption of food and water was observed. If animals exhibited symptoms of reduced body temperature (cool to the touch, shivering), a heating pad was placed under one-half of the cage to offer a warmer environment. Infusion was stopped at once clinical signs of botulism resolved in all rats and tethers were removed. Clinical observations were continued for at least an additional 48 h after treatment cessation to assess for rebound symptoms of botulism.

### Antitoxin efficacy studies

For antitoxin treatments, an anti-BoNT/A sheep polyclonal antitoxin which neutralizes 5,000 LD_50_/µL was generously provided by Dr Shoemaker (Tufts University) (Mukherjee et al. 2012; Tremblay et al. 2020). To ensure rapid termination of BoNT exposure, rats were treated with 4 µL of antitoxin (20,000 neutralizing units) diluted into 250 uL of PBS with 0.05% gelatin by intravenous injection into the tail vein. This antitoxin dose exceeded the toxin challenge dose by 2,000–10,000-fold neutralizing units.

### Semi-quantitative assessment of toxic signs

Three researchers blinded to the groups conducted clinical scoring of toxic signs of botulism using the following scoring rubric [modified from (Vazquez-Cintron et al. (2020)]: (A) respiratory signs: mild abdominal paradox (score of 1), moderate abdominal paradox (3), or severe abdominal paradox and/or agonal respiratory pattern (6); and (B) skeletomuscular signs: salivation (1) and lethargy (1), limb weakness (3), or total body paralysis (lack of righting reflex, 6). Human endpoint criteria included weight loss exceeding 35%, full body paralysis (moribundity) or severe agonal respiratory pattern with rales. Rats reaching euthanasia criteria were given a score of 12 and the time of death was recorded as 12 h later. Rats were given a score of 16 upon death.

### Statistics

Efficacy data represents the cumulative results from at least two separate studies per group. Continuous variables with normal distribution are presented as mean ± SEM and toxic sign scores are presented as median values or median ± interquartile range (IQR). Toxin potency was determined using simple logistical regression. Median survival was determined from Kaplan–Meier survival curves and compared among all treatment groups using Mantel-Cox log-rank test. Pairwise comparisons in median survival time were made against vehicle controls using a Bonferroni-adjusted significance threshold, which was determined by dividing 0.05 by the number of pairwise comparisons. Progression of toxic signs was compared among groups using two-way repeated measures ANOVA with either Sidak’s multiple comparisons test (for two groups) or Tukey’s multiple comparisons test (for more than two groups). Survival outcomes among three or more groups were first compared using the Chi-square test followed by pairwise comparisons against vehicle controls with Fisher’s exact test. Statistical comparisons were made in Prism version 10.3.1 (Graphpad Software, San Diego, CA). Differences were considered significant at the 95% confidence level (*P* < 0.05) except as described above. Additional details on statistical comparisons, normality tests and sample sizes are presented in Supplemental Table 1, individual figure legends and the results section.

## Results

### 3,4-DAP provides therapeutic benefits up to 10 LD50

We previously found that continuous subcutaneous (SQ) infusion of 3,4-DAP at ≥ 1.0 mg/kg•h (producing an estimated steady-state plasma level of 130 ng/mL) has antidotal efficacy in rats challenged with 2.5 LD_50_ BoNT/A (Machamer et al. 2022). The clinical signs of botulism resolved by 14 d after intoxication and withdrawal of infusion did not result in a rebound of clinical signs, although the rats exhibited residual subclinical paralysis, including reductions in endplate potential (EPP) amplitude, miniature EPP frequency and EPP success rate. This steady-state plasma level is within the safe and tolerable clinical exposure, suggesting that 3,4-DAP may be suitable for the treatment of clinical botulism (Thakkar et al. 2017). To further explore the therapeutic potential of 3,4-DAP against the typical range of foodborne doses, survival outcomes were evaluated after challenge with 1.8 LD_50_, 4 LD_50_ and 10 LD_50_ BoNT/A (Pirazzini et al. 2017). In most cases, SQ infusions with vehicle or 3,4-DAP were started once toxic signs appeared in a plurality of rats. Rats were treated with continuous infusions of saline vehicle and 3,4-DAP until clinical signs reached humane endpoint criteria or symptoms resolved fully.

**Fig. 1:**
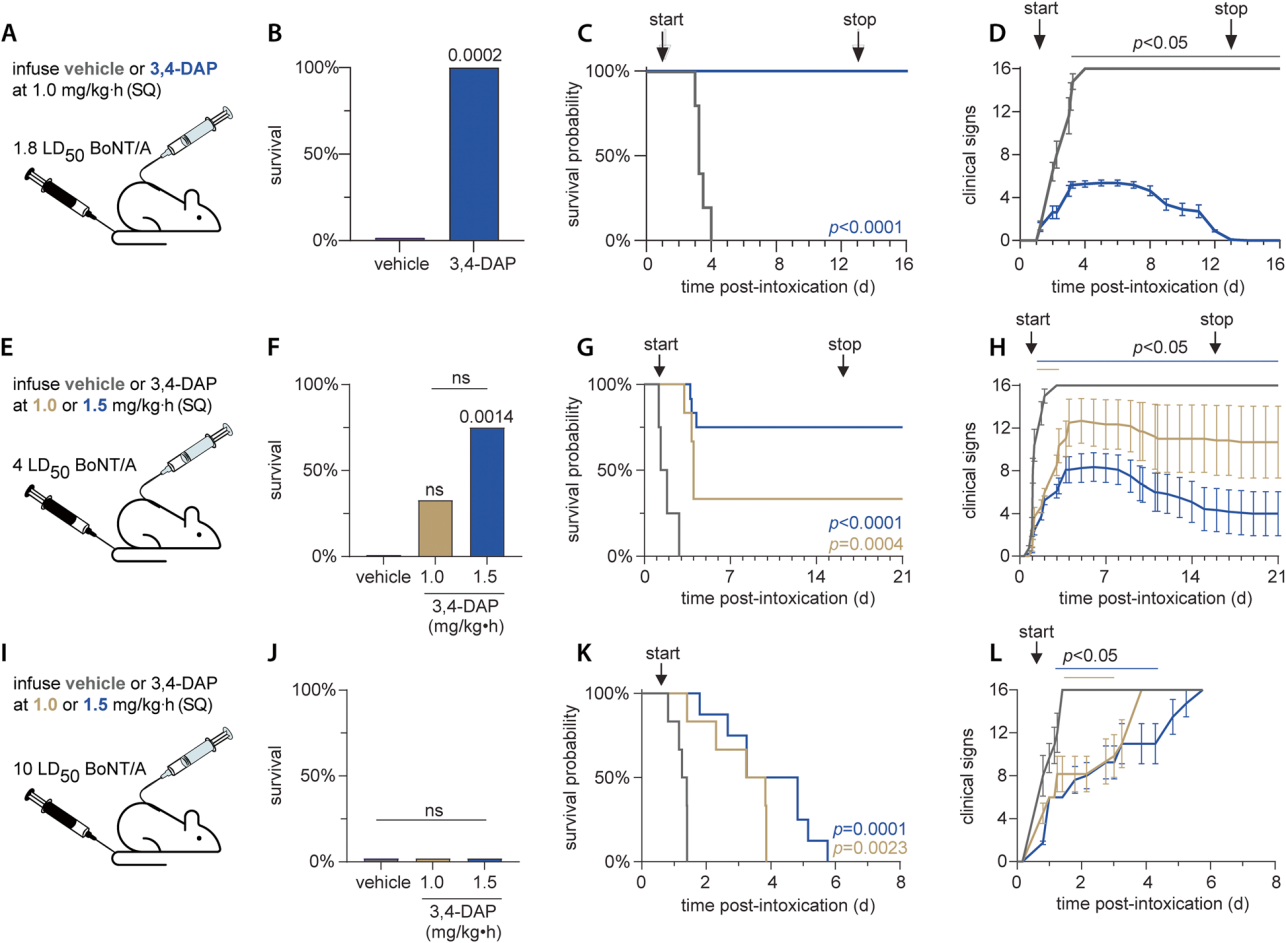
Continuous infusion of 3,4-DAP has therapeutic benefits in rats challenged with up to 10 LD_50_ BoNT/A. **A**, **E**, **I** Cartoons depicting intravenous (IV) BoNT/A challenge followed by subcutaneous (SC) infusion of 1.0 or 1.5 mg/kg•h 3,4-DAP. **B**, **F**, **J** Percent survival for each treatment condition through the end of infusion.* P*-values versus vehicle are presented above each condition. **C**, **G**, **K** Kaplan–Meier survival curves depicting survival proportion over time. *P*-values are color coded and indicate significant prolongation in the median time-to-death (MTD) versus vehicle. The times at which continuous infusion is started and terminated are depicted by arrows above each graph. **D**, **H**, **L** Mean ± SD values for the progression of clinical signs of botulism over time. Animals were not censored at death. The start and stop of continuous infusion are depicted by vertical arrows above each graph. The colored lines above each graph indicate times at which toxic signs were significant different than vehicle (*p* < 0.05) for each treatment condition. Specific details regarding group sizes and statistical analyses are presented in the text and in Supplemental Table 1

3,4-DAP effectively treated clinical signs of botulism after challenge with 1.8 LD_50_ (Fig. 1A). Infusions with saline vehicle (*n* = 5) or 1.0 mg/kg•h 3,4-DAP (*n* = 11) began at 30 h after intoxication, when 50% (8/16) of the rats exhibited clinical signs of systemic botulism. 3,4-DAP had antidotal effects, with 100% survival in the 3,4-DAP infused group versus 0% survival in the vehicle-treated group (*p* = 0.0002; Fig. 1B, C). Toxic signs scores diverged between the treatment groups by 3.25 d and remained significantly different through the end of the study (Fig. 1D). Infusion was stopped at 13 d after intoxication, when the median clinicals signs score fell below 1. Rats did not show rebound of toxic signs and remained healthy and active after withdrawal of treatment (Fig. 1D).

3,4-DAP exhibited partial efficacy after a 4 LD_50_ BoNT/A challenge. Infusions with vehicle (*n* = 8), 1.0 mg/kg•h 3,4-DAP (*n* = 6) or 1.5 mg/kg•h 3,4-DAP (*n* = 12) were started at 24 h after intoxication, when 58% (15/26) of rats presented with clinical signs of botulism (Fig. 1E). Significant differences in survival rates were apparent among the three treatment groups, with improved survival in the 1.5 mg/kg•h 3,4-DAP (9/12 survivors; *p* = 0.0014) versus vehicle (0/8; Fig. 1F). While 33% of rats (2/6) survived in the 1.0 mg/kg•h 3,4-DAP group, this was not a significant improvement over vehicle treatment (*p* = 0.17). Although survival was markedly higher at 1.5 mg/kg•h 3,4-DAP versus 1.0 mg/kg•h 3,4-DAP (75% vs 33%), the difference was not significant (*p* = 0.14), likely due to insufficient statistical power. Compared to the median time to death (MTD) in vehicle-treated rats (1.6 d), MTD was significantly prolonged by treatment with 1.0 mg/kg•h 3,4-DAP (4.0 d; *p* = 0.0004) or 1.5 mg/kg•h 3,4-DAP (> 50% survival;* p* < 0.0001; Fig. 1G). Similar to survival rates, there were no differences in MTD between the two 3,4-DAP treatment groups (*p* = 0.08). However, 1.5 mg/kg•h 3,4-DAP improved clinical signs from 1.2 d through the end of the study, whereas 1.0 mg/kg•h 3,4-DAP merely improved clinical signs from 1.2–3.2 d (Fig. 1H). Although we previously identified a dose-dependent effect of 3,4-DAP infusion on MTD and survival between 0.65 mg/kg•h and 1.0 mg/kg•h (Machamer et al. 2022), the only dose-dependent effects observed here between 1.0 or 1.5 mg/kg•h were in clinical signs, but not survival or MTD.

We next extended the challenge dose to 10 LD_50_ BoNT/A, which is at the upper range of the typical estimated foodborne dose (Pirazzini et al. 2017). Rats were infused (SQ) with vehicle (*n* = 6), 1.0 mg/kg•h 3,4-DAP (*n* = 6), or 1.5 mg/kg•h 3,4-DAP (*n* = 8) starting 16 h after intoxication, when clinical signs of botulism were first detected (Fig. 1I). Despite the lack of survival in all groups (Fig. 1J), MTD was prolong versus vehicle (1.3 d) in rats treated with 1.0 mg/kg•h 3,4-DAP (3.5 d; *p* = 0.0001) or 1.5 mg/kg•h 3,4-DAP (4 d; *p* < 0.0001; Fig. 1K). As with the 4.0 LD50 challenge, there were no differences between the 3,4-DAP treatment groups in survival (*p* > 0.99) or MTD (*p* = 0.13). Clinical signs were improved in rats treated with either dose of 3,4-DAP from approximately 1–3 d (Fig. 1L). Although 3,4-DAP infusion did improve MTD at 10 LD50 BoNT/A challenge, it did not result in antidotal outcomes. In comparison to the 1.8 LD_50_, 4 LD_50_ and previously published 2.5 LD_50_ challenge data (Machamer et al. 2022), these findings confirm our hypothesis that 3,4-DAP efficacy is inversely proportional to the BoNT exposure.

At 1.8 LD_50_ and 4 LD_50_, survivors did not undergo symptomatic rebound when 3,4-DAP infusion was stopped after clinical signs of botulism resolved. Given the rapid clearance of 3,4-DAP in rat (T_1/2_ = 15.9 min; clearance = 7200 mL/kg•h) (Ishida et al. 2019), it is likely that 3,4-DAP is effectively eliminated from the blood within hours after treatment withdrawal. The lack of symptomatic rebound in the absence of 3,4-DAP suggests that the resolution of clinical signs represents a point when sufficient neuromuscular function has recovered to allow survival, despite evidence of residual presynaptic intoxication (Machamer et al. 2022). Of note, the median duration of clinical signs in survivors of the 4 LD50 BoNT/A challenge (16 d, *n* = 11; Fig. 1G) persisted longer than after challenge with 1.8 LD_50_ BoNT/A (12 d; *n* = 11; Fig. 1C) or 2.5 LD_50_ BoNT/A (13 d; *n* = 13) (Machamer et al. 2022), suggesting a positive correlation between toxin dose and duration of muscle paralysis.

### Combination therapy with 3,4-DAP and antitoxin has additive benefits

**Fig. 2:**
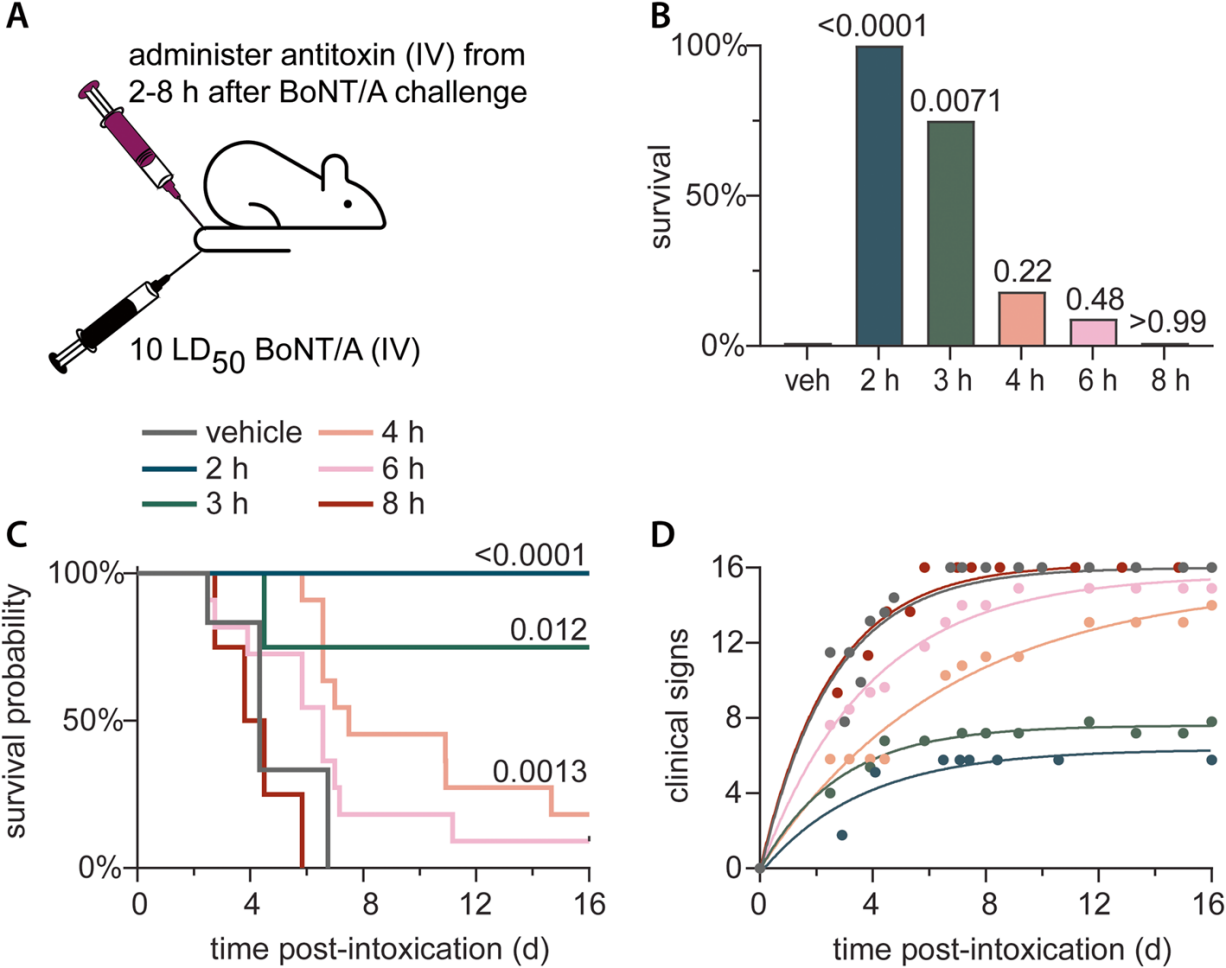
Antitoxin has a narrow treatment window at 10 LD_50_ BoNT/A. **A** Cartoon of experimental strategy. Rats were challenged by IV administration of 10 LD_50_ BoNT/A and treated with IV injection of vehicle at 2 h or neutralizing antitoxin (20,000 units) at 2, 3, 4, 6 or 8 h after BoNT/A challenge. **B** Percent survival for each treatment condition.* P*-values versus vehicle are presented above each condition. **C** Kaplan–Meier survival curves. *P*-values are presented for treatment latencies that prolonged MTD. **D** Mean values and best-fit curves for the progression of clinical signs of botulism over time. Animals were not censored at death. The legend for panels **C** and **D** is above panel **C**. Specific details regarding group sizes and statistical analyses are presented in the text and in Supplemental Table 1

The current standard of care for clinical diagnosis of botulism is infusion of a neutralizing antitoxin. To test the benefits of combined treatment with 3,4-DAP and antitoxin, we used the 10 LD_50_ BoNT/A challenge dose, against which 3,4-DAP prolonged median time-to-death but did not improve survival (Fig. 1J, K). Prior to starting combined treatments, we established treatment delays at which antitoxin was not effective, thus reproducing the most common clinical failure of HBAT therapy. Catheterized rats were challenged with 10 LD_50_ BoNT/A and treated with saline vehicle (IV bolus) after 2 h or with 4 uL antitoxin (IV bolus; corresponding to 20,000 neutralizing units) after 2 h, 3 h, 4 h, 6 h or 8 h (Fig. 2A). In all cases, antitoxin was administered prior to toxic sign emergence, which occurred after 15 h. Delaying antitoxin treatment had a steep effect on survival, with significant differences in survival rates among the six treatment groups (*p* < 0.0001; Fig. 2B). Antitoxin improved survival rates when given within 3 h of intoxication but did not improve survival at 4 h and later (Fig. 2B). MTD was significantly prolonged after a 4 h treatment delay (1.9 d vs vehicle MTD of 1.1 d; *p* = 0.0003) but not after 6 h (1.6 d; *p* = 0.08) or 8 h (1.0 d; *p* = 0.33; Fig. 2C) treatment delays. Toxic signs exhibited significant differences among treatment groups (*p* < 0.0001) although pairwise comparisons were not conducted (Fig. 2D). These data confirm the narrow therapeutic window of antitoxin treatment, which closes before symptom emergence in rats challenged with high doses of BoNT/A.

**Fig. 3:**
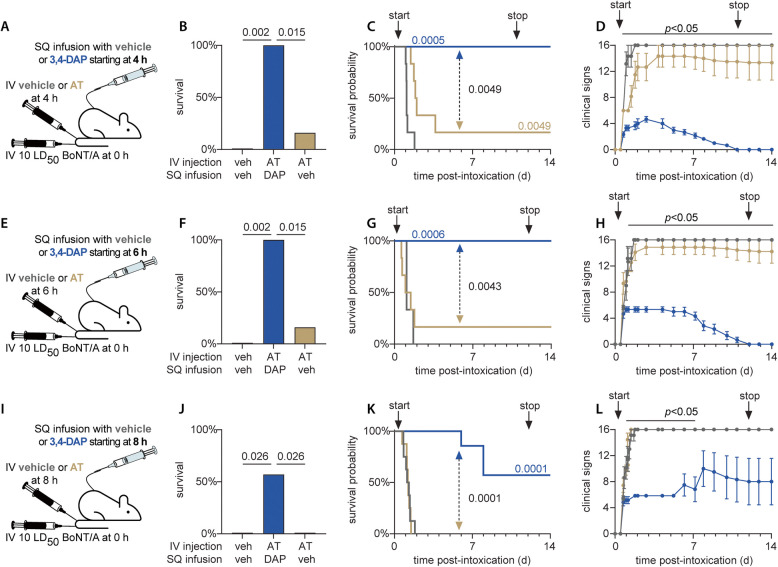
Combination treatment produces significantly improved outcomes versus either treatment. **A**, **E**, **I** Cartoons of experimental strategy. Rats were intoxicated by IV administration of 10 LD_50_ BoNT/A. Rats were then treated starting at 4 h (**A**-**D**), 6 h (**E**–**H**) or 8 h (**I**-**L**) with bolus IV administration of vehicle plus SQ infusion of vehicle; bolus IV administration of 20,000 neutralizing units of antitoxin plus SQ infusion of vehicle; or bolus IV administration of 20,000 neutralizing units of antitoxin plus SQ infusion of 1.0 mg/kg•h 3,4-DAP. **B**, **F**, **J** Percent survival for each treatment condition through the end of infusion.* P*-values are presented for treatment conditions that produced significant improvements in survival. **C**, **G**, **K** Kaplan–Meier survival curves. *P*-values are color coded and indicate significant prolongation of MTD versus vehicle. Black *p*-values indicate statistical comparisons of MTD between antitoxin vs antitoxin plus BoNT/A. **D**, **H**, **L** Mean ± SEM values for the progression of clinical signs of botulism over time. Animals were not censored at death. The black line above each graph indicates time points at which combination treatment significantly improved clinical signs of botulism versus vehicle-treated rats. Specific details regarding group sizes and statistical analyses are presented in the text and in Supplemental Table 1

We next tried a combination treatment approach in which catheterized rats were challenged with 10 LD_50_ BoNT/A and given either: (1) saline vehicle injection plus saline vehicle infusion (SC) starting 2 h after challenge; (2) antitoxin injection plus saline vehicle infusion (aka antitoxin monotherapy) starting 4 h, 6 h or 8 h after challenge; or (3) antitoxin injection combined with 1.0 mg/kg•h 3,4-DAP infusion starting 4 h, 6 h or 8 h after challenge (Fig. 3A). Combination treatment with 3,4-DAP plus antitoxin significantly improved survival versus vehicle or antitoxin monotherapy at all treatment delays (Fig. 3B), with 100% survival at 4 h and 6 h treatment delays (*p* < 0.0001 for each) and 57% survival at 8 h treatment delay (4/7; *p* = 0.026). All three deaths in the 8 h treatment delay group were due to weight loss-related endpoint criteria rather than respiratory failure. There were no survivors in the vehicle-treated groups (0/16), and antitoxin monotherapy did not improve survival rates after 4 h (1/6 survivors; *p* = 0.24), 6 h (1/9 survivors; *p* = 0.32) or 8 h (0/8 survivors; *p* > 0.99) treatment delays. Combined treatment significantly improved MTD at all treatment delays (*p* < 0.0001; Fig. 3C), with significant extension of MTD versus antitoxin monotherapy at 4 h (*p* = 0.0043), 6 h (*p* = 0.0015) and 8 h (*p* = 0.0001). In the combined treatment group, toxic signs stabilized by 1–2 d and fully resolved by 10 d (4 h and 6 h treatment delay) or 11 d (8 h treatment delay). No rebound of toxic signs was observed following treatment withdrawal (Fig. 3D, H and L). Toxic signs for all combination treatment groups were significantly improved compared to vehicle starting at 2 d (Supplemental Table 1). Similar to Fig. 2C, antitoxin treatment alone significantly improved MTD versus vehicle at 4 h (1.9 d vs 1.4 d; *p* = 0.0018) but not at 6 h (*p* = 0.25) or 8 h (*p* = 0.29) treatment delays. These findings confirmed that combination treatment with antitoxin and 3,4-DAP produces survival outcomes that are significantly improved compared to either treatment alone.

### 3,4-DAP provides symptomatic benefits at times that antitoxin is ineffective

**Fig. 4:**
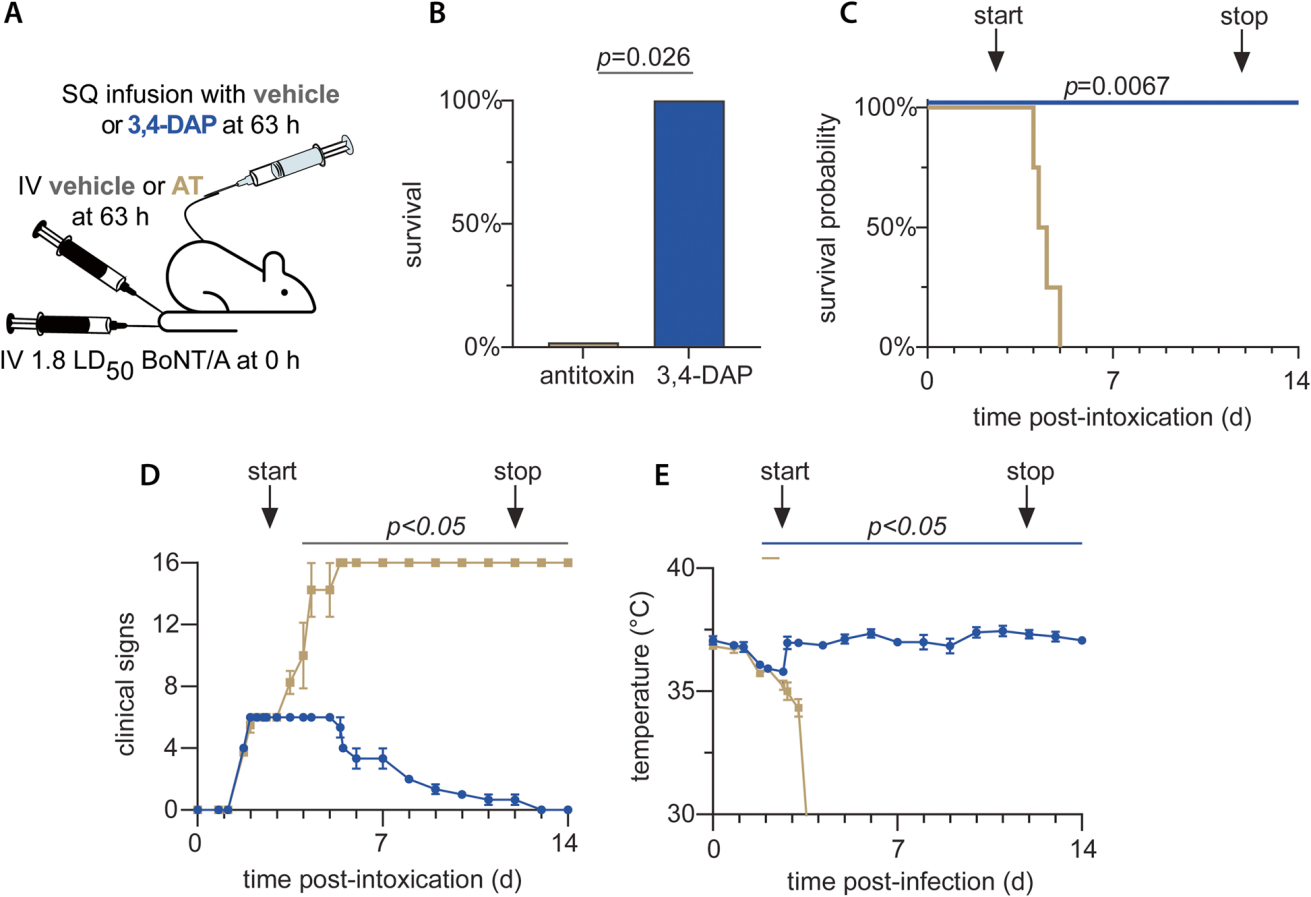
Delayed treatment with 3,4-DAP infusion results in significantly improved outcomes than for antitoxin treatment. **A** Cartoon of experimental strategy. Rats were challenged by IV administration of 1.8 LD_50_ BoNT/A. At 63 h after challenge, rats were treated with SQ infusion of 1.0 mg/kg•h 3,4-DAP (*n* = 4) or with neutralizing antitoxin (20,000 units; IV) plus SQ infusion of saline (*n* = 4). **B** Percent survival for each treatment condition. **C** Kaplan–Meier survival curves. **D** Mean ± SEM values for the progression of clinical signs of botulism over time. Animals were not censored at death. **E** Mean ± SEM temperatures over time. The black line above each graph indicates time points at which 3,4-DAP treatment was different from antitoxin; the plum-colored line represents times at which temperatures in the 3,4-DAP treatment group were significantly different from baseline. Specific details regarding group sizes and statistical analyses are presented in the text and in Supplemental Table 1

The short therapeutic window of antitoxin is a significant limitation to antitoxin therapy. Given the difficulty in diagnosing early-stages of botulism without real-time epidemiological intelligence, the need to request release of antitoxin from regional stockpiles, and the requirement to infuse antitoxin over hours to monitor for hypersensitivity, serum sickness or other adverse infusion reactions (Sobel 2005), there is a clear and urgent need for new therapies that offer longer therapeutic windows. Because 3,4-DAP is a symptomatic treatment that appears effective throughout the course of disease, we hypothesized that delayed treatment with 3,4-DAP would improve survival outcomes. To compare therapeutic windows between antitoxin and 3,4-DAP, rats were intoxicated with 1.8 LD_50_ BoNT/A and treated with 3,4-DAP infusion (1 mg/kg•h, *n* = 4) or saline infusion plus antitoxin injection (IV; 20,000 neutralizing units, *n* = 4) at 63 h post-intoxication (Fig. 4A), when rats exhibited clinical signs of end-stage botulism, including agonal breathing and near whole-body paralysis. Delayed treatment with 3,4-DAP resulted in 100% survival, whereas delayed treatment with antitoxin plus saline infusion resulted in 0% survivors (*p* = 0.026; Fig. 4B, C). Clinical signs stabilized after the start of 3,4-DAP treatment and resolved by 13 d (Fig. 4D). 3,4-DAP treatment produced a significant improved in clinical signs compared to vehicle shortly after the start of treatment that persisted through the remainder of the study, confirming the efficacy of 3,4-DAP at treatment delays that render antitoxin ineffective.

In this experiment, rats were implanted with a temperature monitoring biochip to assess the potential effects of botulism and treatment on core body temperatures. Intoxication led to a progressive decline in body temperature that became significant by 43 h post-intoxication (Fig. 4E). Within 5 h after start of infusion, the body temperature of the 3,4-DAP-treated group rebounded to baseline whereas the body temperature of the antitoxin-treated group continued to decline until death.

## Discussion

We and others have found that 3,4-DAP is a rapidly acting symptomatic treatment for botulism, with sustained administration resulting in antidotal outcomes (Bradford et al. 2018; Machamer et al. 2022; He et al. 2024). Notably, 3,4-DAP is the first small molecule to demonstrate post-symptomatic therapeutic benefit in botulism models. Our finding in rodents also aligns with off-label clinical studies suggesting that bolus treatment with 3,4-DAP or the closely related 4-aminopyridine partially reverses clinical signs of botulism in foodborne patients intoxicated with BoNT serotypes A (Friggeri et al. 2013), B (Dock et al. 2002), or E (Ball et al. 1982).

Here, we further explored the clinical potential of 3,4-DAP across the range of BoNT/A doses typically encountered during foodborne poisoning (1.8–10 LD50 BoNT/A). Continuous subcutaneous infusion of clinically relevant doses of 3,4-DAP significantly improved survival, reversed respiratory paralysis, and alleviated clinical symptoms at doses substantially higher than previously evaluated (Machamer et al. 2022). These infusion rates produced steady-state plasma levels plasma concentrations deemed safe in clinical practice (Thakkar et al. 2017). Furthermore, we demonstrated additive therapeutic efficacy when 3,4-DAP was combined with antitoxin treatment. Importantly, delayed administration of 3,4-DAP provided survival benefits at times when antitoxin therapy was no longer effective. Collectively, these findings confirm that 3,4-DAP provides both adjunctive and stand-alone benefits as a treatment for botulism. These findings validate and extend prior preclinical studies and position 3,4-DAP as a repurposed therapeutic to mitigate the morbidity and mortality associated with botulism.

Although antitoxin typically does not prevent respiratory arrest when given to symptomatic patients, it can decrease the duration of paralysis by blocking additional uptake of BoNT (Richardson et al. 2019; Yu et al. 2017). Given the inverse relationship between BoNT/A dose and 3,4-DAP efficacy, we hypothesized improved therapeutic outcomes when 3,4-DAP was combined with antitoxin, due to reduced toxin exposure. Because the therapeutic mechanism of 3,4-DAP is distinct from that of antitoxin, the risks of adverse drug interactions are low. Indeed, we observed that combination therapy approach showed a significantly extended therapeutic window and substantially improved survival compared to either treatment alone, especially when treatment was delayed beyond the therapeutic window for antitoxin monotherapy. This combinatorial approach consistently provided additive benefits, particularly evident at longer treatment delays when antitoxin monotherapy had no impact on survival. This combined regimen may thus reduce morbidity and healthcare costs by accelerating recovery, shortening intensive supportive care, and minimizing ventilator-associated complications(Arnon et al. 2006; Souayah et al. 2012; Anderson et al. 2019).

The symptomatic efficacy of 3,4-DAP persisted even when treatment initiation occurred well beyond the therapeutic window of antitoxin. In late-stage botulism characterized by severe respiratory distress and advanced neuromuscular paralysis, delayed treatment with 3,4-DAP rapidly reversed clinical signs and resulted in complete survival, whereas delayed treatment with antitoxin alone provided no benefit. The extended therapeutic window of 3,4-DAP is clinically important, particularly in scenarios involving delayed diagnosis or logistical challenges to rapid antitoxin administration, such as in rural areas or during mass casualty events. Given the known logistical barriers to rapid antitoxin access in clinical practice (Sobel 2005), these findings highlight the therapeutic potential of 3,4-DAP as adjunctive treatment with antitoxin, facilitating rapid respiratory stabilization and survival until natural recovery occurs.

The observed inverse relationship between toxin dose and 3,4-DAP efficacy aligns with the proposed therapeutic mechanism of 3,4-DAP. At neuromuscular junctions, a small fraction (~ 5%) of docked and primed synaptic vesicles release acetylcholine in response to neuronal depolarization (Tarr et al. 2013; Ruiz et al. 2011; Laghaei et al. 2018). Vesicle fusion depends on the Ca^2+^-triggered activation of SNARE protein release complexes containing SNAP-25, synaptobrevin, and syntaxin (Poirier et al. 1998; Sutton et al. 1998; Hanson et al. 1997). BoNT-induced cleavage of SNARE proteins disrupts these complexes, progressively reducing acetylcholine release (Beske et al. 2017; Bradford et al. 2018; Lu 2015; Hayashi et al. 1994; Khounlo et al. 2017). By blocking potassium channels and prolonging presynaptic depolarization, 3,4-DAP increases the probability of acetylcholine release from vesicle release sites that are not associated with cleaved SNARE proteins, thereby partially reversing toxin-induced paralysis (Bradford et al. 2018; Ng et al. 2017). This mechanism likely explains why 3,4-DAP is less effective as toxin load increases: there are fewer intact vesicle release sites available to undergo membrane fusion and acetylcholine release. Notably, SNAP-25 cleavage by BoNT/A produces SNAP-25(1–197), which retains residual fusogenic activity (Lu 2015; Khounlo et al. 2017), potentially explaining the serotype-specific enhanced efficacy of 3,4-DAP against BoNT/A compared to other serotypes (Beske et al. 2017, 2018; Bradford et al. 2018; Vazquez-Cintron et al. 2020). Future studies quantifying therapeutic effects after exposure to other BoNT serotypes are required to confirm this hypothesis.

Additionally, this study provides novel evidence that core body temperature is a reliable biomarker of intoxication progression and therapeutic efficacy. Implanted temperature-monitoring biochips revealed significant hypothermia preceding overt clinical symptoms, consistent with our ad hoc observations of reduced body temperatures in 3,4-DAP-treated rats. Hypothermia likely arises from impaired peripheral vasoconstriction (Goldberg et al. 2023), reduced metabolic heat production, and diminished shivering secondary to muscle paralysis (Gordon 1990), all of which may be directly or indirectly affected by botulism paralysis. Furthermore, we have reported decreased ventilation, increased blood CO_2_ levels (hypercapnia) and respiratory acidosis in a mouse botulism model (McClintic et al. 2024), which have been previously shown to cause acute declines in body temperature in rats (Granjeiro et al. 2016). The rapid restoration of normal body temperature following 3,4-DAP treatment further emphasizes its swift symptomatic relief and suggests a new clinical indicator for monitoring intoxication severity and treatment efficacy.

A critical factor influencing clinical translation of novel therapeutics is the feasibility of drug administration routes. We previously reported that the short half-life of 3,4-DAP in rodents (~ 15 min) resulted in an ephemeral effect on botulism symptoms in vivo when 3,4-DAP was administered as a bolus injection (Vazquez-Cintron et al. 2020; Machamer et al. 2022). To compensate for the rapid clearance of 3,4-DAP, we developed an infusion model to test continuous 3,4-DAP treatment on botulism symptoms while avoiding the troughs and peaks of bolus administration. Consistent with our previous work, the infusion dose rates used here did not produce any evidence of tolerance, functional toxicity or apparent loss of efficacy throughout the infusion period. Indeed, continuous subcutaneous infusion of 3,4-DAP provided robust therapeutic effects at clinically relevant infusion rates (Thakkar et al. 2017). Although these studies used subcutaneous administration, intravenous (IV) infusion of 3,4-DAP may ultimately be preferred for clinical studies. IV administration offers greater control over plasma drug levels, which will be important if the therapeutic index of 3,4-DAP proves narrow. Furthermore, botulism patients typically receive intravenous hydration, enabling convenient IV administration of 3,4-DAP. Currently, 3,4-DAP is formulated as an oral tablet administered multiple times daily; however, botulism symptoms persist for prolonged periods, and we observed rapid symptom rebound and death after premature treatment cessation (Vazquez-Cintron et al. 2020; Machamer et al. 2022). Continuous IV administration allows administration of a wider range of 3,4-DAP doses while reducing the risk of adverse neurological effects and symptom breakthrough during pharmacokinetic trough periods. As IV delivery of 3,4-DAP has not yet been clinically evaluated, additional preclinical studies assessing intravenous pharmacokinetics, safety, tolerability, and optimal infusion protocols are required.

In summary, this study provides robust preclinical support for clinical evaluation of 3,4-DAP as a symptomatic treatment for botulism and, by extension, raises the possibility that continuous infusion of 3,4-DAP can offer benefits for other diseases involving reduced acetylcholine release. Given its FDA approval and established safety profile in treating neuromuscular disorders, rapid clinical translation of 3,4-DAP appears feasible. Future investigations should address efficacy against additional BoNT serotypes, potential synergy with emerging intracellular antidotes, and applicability against other toxins causing respiratory paralysis.

## Supplementary Information

Supplementary Material 1: Table S1. Full details of experimental parameters, group sizes and statistical tests.

## Data Availability

Data are provided within the manuscript or supplementary information files. Any additional requests will be accommodated by the corresponding author upon reasonable request.
