# Supplementary material for: Combined treatment with antitoxin and 3,4-diaminopyridine improves survival outcomes after lethal botulinum neurotoxin challenge
Source: Mol Med. 2025 Jul 28;31:270. doi: 10.1186/s10020-025-01316-0 (PMC12305929; doi:10.1186/s10020-025-01316-0)
Supplement: Supplementary file 1 — Supplementary Material 1: Table S1. Full details of experimental parameters, group sizes and statistical tests. [file 10020_2025_1316_MOESM1_ESM.docx]

**Table S1. Summary of studies, conditions and statistical comparisons**

| **Figure** | **Conditions**  **sample size (n), number of studies (ns)** | **Compared value** | **Primary test** | **Primary test result** | **Secondary test** | **Secondary test result** |
| --- | --- | --- | --- | --- | --- | --- |
| **Figure 1A-D** Challenge: 1.8 LD_50_  Comparisons:  - vehicle  - 1.0 mg/kg•h 3,4-DAP  - 30 h treatment latency | saline vehicle: n=5, ns=2  1.0 mg/kg•h 3,4-DAP: n=11, ns=2 | survival^1^ | Fisher’s exact test | vehicle (0/5) *vs* 1.0 mg/kg•h (11/11): ***p*=0.0002** |  |  |
|  |  | median survival time | Mantel-Cox log-rank test | χ^2^=19.6, ***p*<0.0001** | pairwise Mantel-Cox log-rank test | vehicle (3.25 d) *vs* 3,4-DAP (>50% survival): ***p*<0.0001** |
|  |  | clinical signs | two-way repeated measures ANOVA | time x treatment: *F*(20,280)=81.7, ***p*<0.0001** | Šídák's multiple comparisons test | 3,4-DAP significantly different from vehicle starting at **3.1 d** |
| **Figure 1E-H** Challenge: 4 LD_50_  Treatments:  - vehicle  - 1.0 mg/kg•h 3,4-DAP  - 1.5 mg/kg•h 3,4-DAP  - 24 h treatment latency | saline vehicle: n=8, ns=2  1.0 mg/kg•h 3,4-DAP: n=6, ns=2  1.5 mg/kg•h 3,4-DAP: n=12, ns=2 | survival^1^ | Chi-square test | χ^2^=11.3, ***p*=0.0035** | Fisher’s exact test | vehicle (0/8) *vs* 1.0 mg/kg•h (2/6): *p*=0.17  vehicle (0/8) *vs* 1.5 mg/kg•h (9/12): ***p*=0.0014**  1.0 mg/kg•h (2/6) *vs* 1.5 mg/kg•h (9/12): p=0.14 |
|  |  | median survival time | Two-sided Fisher’s exact test | vehicle (0/8) *vs* all DAP treatments (11/18): ***p*=0.0074** |  |  |
|  |  |  | Mantel-Cox log-rank test | χ^2^=34.2, ***p*<0.0001** | pairwise Mantel-Cox log-rank test | vehicle (1.6 d) *vs* 1.0 mg/kg•h (4 d): ***p*=0.0004**  vehicle (1.6 d) *vs* 1.5 mg/kg•h (>50% survival): ***p*<0.0001**  1.0 mg/kg•h (4 d) *vs* 1.5 mg/kg•h (>50% survival): p=0.083 |
|  |  | clinical signs | two-way repeated measures ANOVA | time x treatment: *F*(62,713)=5.8, ***p*<0.0001** | Tukey's multiple comparisons test | 1.0 mg/kg•h 3,4-DAP significantly different from veh from **1.2 to 3.2 d**  1.5 mg/kg•h 3,4-DAP significantly different from veh starting at **1.2 d**  1.0 mg/kg•h 3,4-DAP not different from 1.5 mg/kg•h 3,4-DAP |
| **Figure 1I-L**  Challenge: 10 LD_50_  Treatments:  - vehicle  - 1.0 mg/kg•h 3,4-DAP  - 1.5 mg/kg•h 3,4-DAP  - 16 h treatment latency | saline vehicle: n=6, ns=3  1.0 mg/kg•h 3,4-DAP: n=6, ns=2  1.5 mg/kg•h 3,4-DAP: n=8, ns=2 | survival^1^ | Chi-square test | no result: 100% mortality |  |  |
|  |  | median survival time | Mantel-Cox log-rank test | χ^2^=23.2, ***p*<0.0001** | pairwise Mantel-Cox log-rank test | vehicle (1.3 d) *vs* 1.0 mg/kg•h (3.5 d): ***p*=0.0023**  vehicle (1.3 d) *vs* 1.5 mg/kg•h (4 d): ***p*=0.0001**  1.0 mg/kg•h (3.5 d) *vs* 1.5 mg/kg•h (4 d): *p*=0.13 |
|  |  | clinical signs | two-way repeated measures ANOVA | time x treatment: *F*(32,272)=4.0, ***p*<0.0001** | Tukey's multiple comparisons test | 1.0 mg/kg•h 3,4-DAP significantly different from veh from **1.2 to 3.2 d**  1.5 mg/kg•h 3,4-DAP significantly different from veh from **1.4 to 3.2 d**  1.0 mg/kg•h 3,4-DAP not different from 1.5 mg/kg•h 3,4-DAP |
| **Figure 2**  Challenge: 10 LD_50_  Treatments:  - vehicle  - antitoxin  - 2-8 h treatment latency | saline vehicle: n=12, ns=4  2 h antitoxin latency: n=9, ns=2  3 h antitoxin latency: n=4, ns=1  4 h antitoxin latency: n=11, ns=2  6 h antitoxin latency: n=11, ns=2  8 h antitoxin latency: n=4, ns=2 | survival^1^ | Chi-square test | χ^2^=35.1, ***p*<0.0001** | Fisher’s exact test | vehicle (0/12) *vs* 2 h latency (9/9): ***p*<0.0001**  vehicle (0/12) *vs* 3 h latency (3/4): ***p=*0.0071**  vehicle (0/12) *vs* 4 h latency (2/11): *p*=0.22  vehicle (0/12) *vs* 6 h latency (1/11): *p*=0.48  vehicle (0/12) *vs* 8 h latency (0/4): *p*>0.99 |
|  |  | median survival time | Mantel-Cox log-rank test | χ^2^=42.2, ***p*<0.0001** | pairwise Mantel-Cox log-rank test | vehicle (1.1 d) *vs* 2 h latency (<50% survival): ***p*<0.0001**  vehicle (1.1 d) *vs* 3 h latency (<50% survival): ***p=*0.012**  vehicle (1.1 d) *vs* 4 h latency (1.9 d): ***p*=0.0013**  vehicle (1.1 d) *vs* 6 h latency (1.7 d): *p*=0.17  vehicle (1.1 d) *vs* 8 h latency (1.0 d): *p*=0.25 |
|  |  | clinical signs | two-phase nonlinear regression | R^2^ values  vehicle: 0.82  2 h: 0.78  3 h: 0.20  4 h: 0.51  6 h: 0.54  8 h: 0.80 |  |  |
| **Figure 3**  Challenge: 10 LD_50_  Treatments:  - vehicle  - antitoxin  - 1.0 mg/kg•h 3,4-DAP+antitoxin  - 4-8 h treatment latency | 4 h treatment latency (ns=2)  saline vehicle: n=6  antitoxin latency: n=6  antitoxin+3,4-DAP latency: n=6 | survival^1^ | Chi-square test | χ^2^=14.5, ***p*=0.0007** | Fisher’s exact test | vehicle (0/6) *vs* antitoxin+3,4-DAP (6/6): ***p*=0.0022**  antitoxin (1/6) *vs* 3,4-DAP+antitoxin (6/6): ***p*=0.015**  vehicle (0/6) *vs* antitoxin (1/6): *p*>0.99 |
|  |  | median survival time | Mantel-Cox log-rank test | χ^2^=22.0, ***p*<0.0001** | pairwise Mantel-Cox log-rank test | vehicle (1.1 d) *vs* antitoxin+3,4-DAP (>50% survival): ***p*=0.0005**  antitoxin (1.9 d) *vs* 3,4-DAP+antitoxin (>50% survival): ***p*=0.0043**  vehicle (1.1 d) *vs* antitoxin (1.9 d): ***p*=0.0049** |
|  |  | clinical signs | two-way repeated measures ANOVA | time x treatment: *F*(38,285)=12.6, ***p*<0.0001** | Tukey's multiple comparisons test | combined treatment is improved vs antitoxin starting at 0.75 d |
|  | 6 h treatment latency (ns=2)  saline vehicle: n=6  antitoxin+3,4-DAP latency: n=6  antitoxin latency: n=6 | survival^1^ | Chi-square test | χ^2^=14.5, ***p*=0.0007** | Fisher’s exact test | vehicle (0/6) *vs* antitoxin+3,4-DAP (6/6): ***p*=0.0022**  antitoxin (1/6) *vs* 3,4-DAP+antitoxin (6/6): ***p*=0.015**  vehicle (0/6) *vs* antitoxin (1/6): *p*>0.99 |
|  |  | median survival time | Mantel-Cox log-rank test | χ^2^=11.6, ***p*=0.0031** | pairwise Mantel-Cox log-rank test | vehicle (1.1 d) *vs* antitoxin+3,4-DAP (>50% survival): ***p*=0.0006**  antitoxin (1.2 d) *vs* 3,4-DAP+antitoxin (>50% survival): ***p*=0.0043**  vehicle (1.1 d) *vs* antitoxin (1.2 d): *p*=0.83 |
|  |  | clinical signs | two-way repeated measures ANOVA | time x treatment: *F*(42,315)=11.4, ***p*<0.0001** | Tukey's multiple comparisons test | combined treatment is improved vs antitoxin starting at 1.5 d |
|  | 8 h treatment latency (ns=3)  saline vehicle: n=8  antitoxin+3,4-DAP latency: n=7  antitoxin latency: n=8 | survival^1^ | Chi-square test | χ^2^=11.1, ***p*=0.004** | Fisher’s exact test | vehicle (0/8) *vs* antitoxin+3,4-DAP (4/7): ***p*=0.026**  antitoxin (0/8) *vs* 3,4-DAP+antitoxin (4/7): ***p*=0.026**  vehicle (0/8) *vs* antitoxin (0/8): *p*>0.99 |
|  |  | median survival time | Mantel-Cox log-rank test | χ^2^=18.0, ***p*=0.0001** | pairwise Mantel-Cox log-rank test | vehicle (1.2 d) *vs* antitoxin+3,4-DAP (>50% survival): ***p*=0.0001**  antitoxin (1.2 d) *vs* 3,4-DAP+antitoxin (>50% survival): ***p*=0.0001**  vehicle (1.2 d) *vs* antitoxin (1.2 d): *p*=0.87 |
|  |  | clinical signs | two-way repeated measures ANOVA | time x treatment: *F*(40,400)=4.6, ***p*<0.0001** | Tukey's multiple comparisons test | combined treatment is improved vs antitoxin from 1.0-7.0 d |
| **Figure 4**  Challenge: 1,8 LD_50_  Treatments:  - saline  - antitoxin (AT)  - 1.0 mg/kg•h 3,4-DAP  - 63 h treatment latency | saline injection+DAP infusion: n=4, ns=2  AT injection+saline infusion: n=4, ns=2 | survival^1^ | Fisher’s exact test | antitoxin+saline (0/4) vs 3,4-DAP (4/4): ***p*=0.029** |  |  |
|  |  | median survival time | Mantel-Cox log-rank test | vehicle (4.3 d) *vs* 3,4-DAP (>50% survival): χ^2^=7.3, ***p*=0.0067** |  |  |
|  |  | clinical signs | two-way repeated measures ANOVA | time x treatment: *F*(23,138)=95.1, ***p*<0.0001** | Šídák's multiple comparisons test | groups were significantly starting at 4 d |
|  |  | temperatures^2^ | two-way repeated measures ANOVA | time x treatment: *F*(19,114)=1219, ***p*<0.0001** | Šídák's multiple comparisons test | groups were significantly starting at 2.7 d |

Note 1: survival is defined as survival for at least 48 h after (a) withdrawal of treatment or (b) resolution of clinical signs

Note 2: temperatures were reported as 21º C after death
